# Novel Monoclonal Antibody and Peptide Binders for *Mycobacterium avium* subsp. *paratuberculosis* and Their Application for Magnetic Separation

**DOI:** 10.1371/journal.pone.0147870

**Published:** 2016-01-27

**Authors:** Lorna M. O’Brien, Linda D. Stewart, Sam A. J. Strain, Irene R. Grant

**Affiliations:** 1 Institute for Global Food Security, School of Biological Sciences, Queen’s University Belfast, Medical Biology Centre, 97 Lisburn Road, Belfast, BT9 7BL, Northern Ireland, United Kingdom; 2 Animal Health and Welfare Northern Ireland, 97 Moy Road, Dungannon, Co.Tyrone, BT71 7DX, Northern Ireland, United Kingdom; Cornell University, UNITED STATES

## Abstract

The generation of novel *Mycobacterium avium* subsp. *paratuberculosis* (MAP)-specific monoclonal antibodies and phage-display derived peptide binders, along with their application for the magnetic separation (MS) of MAP cells, is described. Our aim was to achieve even greater MAP capture capability than is possible with peptide-mediated magnetic separation (PMS) using a 50:50 mix of biotinylated-aMp3 and biotinylated-aMptD peptide-coated beads. Gamma-irradiated whole MAP cells and ethanol extracted antigens (EEA) from these cells were used to elicit an immune response and as phage-display biopanning targets. A range of novel binders was obtained and coated onto paramagnetic beads, both individually and in various combinations, for MS evaluation. IS900 PCR was employed after MS to provide quick results. Capture sensitivity was assessed using a range of MAP concentrations after which the most promising beads were tested for their specificity for MAP, by performing MS followed by culture using 10 other *Mycobacterium* species. Magnetic beads coated with the biotinylated EEA402 peptide demonstrated a greater level of MAP capture than the current PMS method, even when low numbers of MAP (<10 cfu/ml) were present; however these beads also captured a range of other mycobacteria and so lacked capture specificity. Magnetic beads coated with monoclonal antibodies 6G11 and 15D10 (used as a 50:50 mix or as dually coated beads) also demonstrated improved MAP capture relative to the current PMS method, but with little cross-reactivity to other *Mycobacterium* spp. Therefore, two new MS protocols are suggested, the application of which would be dependent upon the required endpoint. Biotinylated EEA402-coated beads could potentially be used with a MAP-specific PCR to ensure detection specificity, while beads coated with 6G11 and 15D10 monoclonal antibodies could be used with culture or the phage amplification assay.

## Introduction

*Mycobacterium avium* subsp. *paratuberculosis* (MAP) is the causative agent for Johne’s disease, a highly-infectious wasting disease that affects a range of domestic ruminants including cattle, sheep, goats and deer [1]. Johne’s disease presents as a wasting disease which can include persistent diarrhoea, sub-mandibular oedema, and progressive emaciation, ultimately leading to death or premature culling of the infected animal [2]. Some infected animals may be asymptomatic and not show any clinical signs of infection, however both symptomatic and asymptomatic animals can shed MAP in their milk and faeces, thereby constituting an infectious risk to susceptible animals which typically acquire infection through the ingestion of MAP or MAP contaminated material [3, 4, 5]. Often, these sub-clinical animals outnumber clinically infected animals within a herd, and so their rapid identification is key to controlling within-herd transmission of Johne’s disease [4]. ELISA tests based on the detection of MAP-specific antibody in blood or milk samples are the most frequently used tool to detect MAP infection internationally [6]. However, the assay sensitivities of these ELISA-based tests are poor and have been estimated to be approximately 20%, therefore limiting their effectiveness to detect infected animals [7, 8]. Liquid and solid culture is still widely considered as the ‘gold standard’ method for the detection of MAP in bovine milk and faeces, despite the fact that no truly selective media for MAP currently exists [9]. The slow-growing nature of MAP means that chemical decontaminants are often employed to reduce the risk of culture overgrowth by contaminating organisms, which in turn also impairs the viability of MAP to a degree, thereby further reducing the sensitivity of culture. The detection sensitivity of faecal culture has been estimated to be approximately 23%, and therefore often results in large numbers of false negative results. In order to avoid this, molecular-based methods have commonly been employed for the detection of MAP [7, 10, 11]. While these methods are much more rapid and often more sensitive, they are typically unable to assess the viability of the MAP cells, which is important when identifying the infection status of an animal or herd.

Therefore, there is a need for a rapid detection method for MAP which is both specific and sensitive and which can distinguish between viable and non-viable cells. One method which appears to have this capability is the combined PMS (peptide-mediated magnetic separation)-phage assay which has previously been described [12]. PMS involves the isolation of MAP cells using paramagnetic beads coated with the MAP-specific ligands, biotinylated aMp3 and aMptD peptides which were previously identified using phage display biopanning against whole MAP cells [13] and the cell surface peptide (MptD) expressed by the MAP–specific ABC Transporter operon (*mpt*) [14], respectively. A phage amplification assay, optimised previously for MAP [15] is then used to enumerate viable MAP cells only. This PMS-phage assay combination has been successfully applied to the detection of MAP from naturally-contaminated bovine milk and faecal samples [12] and has been found to be 99% specific for MAP and possess a capture efficiency of greater than 85% [16].

This study aims to enhance the efficiency of the MS method further, by improving assay sensitivity through the generation of novel MAP-specific monoclonal antibodies and phage display-derived peptides. Previous studies aimed at generating MAP-specific antibodies often used heat treatments to inactivate MAP cells prior to immunisation [17]. However, in order to maintain an intact MAP cell surface, gamma irradiation was employed in this study. This was previously shown to be successful at generating polyclonal antibodies against MAP and was also the approach used in previous studies relating to *M*. *bovis* monoclonal antibody generation and phage display biopanning [18, 19]. The novel MAP binders were covalently bound to paramagnetic beads and assessed for their ability to isolate MAP cells from cultures where low numbers of MAP are present, in the hope of identifying those which are more sensitive than the current aMp3 and aMptD peptides. The optimised magnetic separation method could then be combined with various endpoints such as culture, PCR or phage amplification assays, depending on the intended application.

## Materials and Methods

### Preparation of immunogen

Immunogens for antibody production were prepared as either whole cell antigens (WCA) or ethanol extracted antigens (EEA) from MAP strain B4 (a Northern Ireland bovine field isolate) which had been grown to stationary phase in Middlebrook 7H9 broth supplemented with 10% OADC (both from Difco™), 0.005% Tween 80 and 2 μg/ml mycobactin J (Synbiotics Europe SAS, Lyon, France). The culture was centrifuged, washed twice in phosphate buffered saline (PBS, pH 7.4) and re-suspended in an equal volume of PBS. The MAP cell concentration was calculated to be ~10^8^ cfu/ml; quantified using the phage amplification assay prior to gamma irradiation to a dose of 10 kGy using a Gammabeam 650 irradiator. The irradiated suspension served as WCA. EEA was generated from the WCA as described by Eda *et al*. [20]. Briefly, irradiated MAP cells (WCA) were vortexed at high speed in 80% (v/v) ethanol solution for approx. 30 sec. The cells were removed through centrifugation, and the supernatant was transferred to a fresh tube. The ethanol was aspirated under nitrogen gas and the protein suspension freeze-dried to remove any excess water before final resuspension in sterile water at a concentration of 1 mg/ml. Both immunogens were stored at -80°C until required.

### Antibody production

All animal procedures were carried out in the Biological Services Unit at Queen’s University Belfast, in accordance with the Animal (Scientific Procedures) Act 1986, under the terms of a project licence issued by the Department of Health, Social Services and Public Safety (Northern Ireland) and with ethical approval granted by the University Animal Welfare and Ethical Review Body. Monoclonal antibodies (MAb) were produced by immunising six Balb/C female mice with either 10^8^ cfu/ml WCA or 1 mg/ml of EEA. For the primary immunisation and first booster immunisation, 50 μl of either WCA or EEA were mixed in Quil A adjuvant (BrennTag, Superfoss, Denmark) and administered subcutaneously at two separate injection sites per mouse. The remaining two immunisations were mixed with Pam3Cys-Ser (Lys) 4-OH (PCSL) adjuvant (EMC Microcollections, GmbH, Germany) and administered intraperitoneally at two injection sites per mouse. All immunisations were administered at 4 week intervals, with blood samples collected from the tail vein of each mouse 10 days post-immunisation. Serum antibodies from each bleed were tested for specific binding to irradiated WCA of MAP B4 using a direct ELISA. The mouse which showed the greatest immunological response following the fourth immunisation with each of the immunogens was selected for B-cell fusion, and received a final booster immunisation (same as last immunisation) four days prior to spleen harvest. Mice were sacrificed by asphyxiation under carbon dioxide. For B-cell fusion, a suspension of spleenocytes was fused with SP2 myeloma cells (modified from [21]) using polyethylene glycol. Hybridoma supernatant was collected and screened using the direct ELISA protocol (described below). Supernatants that were positive by direct ELISA were tested using a competitive ELISA (also described below) to confirm binding to MAP cells. Positive hybridoma cell lines were cloned twice before amplification in triple-layered flasks (see antibody purification protocol below).

### Direct ELISA (screening assay)

Hybridoma supernatant was collected and tested by ELISA for monoclonal antibodies capable of binding whole MAP cells. MaxiSorp™ microtiter plates (Nunc™, Thermo Scientific) were coated with 100 μl/well of 10^8^ cfu/ ml of irradiated WCA of MAP B4 diluted 1/10,000 (v/v) in 0.1 M bicarbonate buffer pH 9.4–9.7. The plates were refrigerated overnight to facilitate antigen binding. Skimmed milk powder (Marvel, 0.5% w/v) dissolved in 1 mM sodium acetate (NaAc) buffer (pH 7.2) was used as a blocking agent, where 300 μl was added to each well and incubated at 37°C for 2 hours. The blocking buffer was discarded and 100 μl of hybridoma supernatant diluted 1:1 (v/v) in NaAc buffer was added to each well, before incubation at 37°C with shaking for 1.5 hours. Foetal bovine serum- free DMEM hybridoma growth media diluted 1:1 (v/v) in NaAc buffer was used as a supernatant negative control. Following this, the supernatant was removed and the plate washed ten times with wash buffer (9 g/l (w/v) NaCl and 0.0125% (v/v) Tween 20 (both Sigma-Aldrich, UK) in deionised water). Horseradish peroxidase conjugated anti-mouse antibody (Dako, Cambridge, United Kingdom) diluted 1/2000 (v/v) in blocking buffer was added (100 μl) to each well and incubated with shaking at 37°C for 1 hour. The plate was washed ten times with wash buffer and 100 μl of 3, 3 =, 5, 5 = -tetramethylbenzidine solution (TMB/E, EMD Millipore Corporation, USA) was added to each well. The colour development was stopped using 25 μl of 2.5 M sulphuric acid and the plate was read at an absorbance value of 450 nm. Serum collected from the final cardiac puncture from the immunised mouse was used as a positive control and fetal bovine serum-free culture media as a negative control. An optical density (OD450nm) reading was considered to be indicative of MAP binding if a value of > 1 was achieved after normalisation of the absorbance values by subtraction of the negative control OD value.

### Competitive ELISA

Hybridoma supernatants with a positive reading on the direct ELISA assay were tested for MAP binding using a competitive ELISA, whereby 100 μl of 10^8^ cfu/ml of WCA MAP B4, diluted 1/10,000 (v/v) in 0.1 M bicarbonate buffer pH 9.4–9.7, was used to coat MaxiSorp™ microtitre plates (Nunc™, Thermo Scientific). The plates were refrigerated overnight to facilitate antigen binding. Skimmed milk powder (Marvel, 0.5% w/v) dissolved in 1 mM NaAc buffer (pH 7.2) was used as a blocking agent, where 300 μl was added to each well and incubated at 37°C for 2 h. The blocking buffer was discarded and 50 μl of 10^8^ cfu/ml of irradiated MAP B4 was added to selected wells. To this, 50 μl of diluted hybridoma supernatant (1:10 (v/v) in NaAc buffer) was added, followed by incubation at 37°C with shaking for 1.5 hours. Negative control wells (wells containing no ‘free MAP cells’) were also included and received 50 μl NaAc buffer and 50 μl of the diluted supernatant (1:10 (v/v) in NaAc buffer). The remainder of the ELISA process was as described above for the direct ELISA. An optical density value which was lower in the presence of the ‘free’ cells, than that seen in the negative control wells demonstrated binding to the ‘free’ MAP WCA.

### Monoclonal antibody purification

Two cell lines (6G11 and 15D10) producing monoclonal antibodies capable of binding whole cells of MAP by both direct and competitive ELISA were cloned twice until a stable cell line was achieved in which all sub-clones produced the anti-MAP monoclonal antibody. Following this, the selected cell lines were grown to maximum confluence in SP2 growth media (DMEM medium containing 10% (v/v) fetal bovine serum and 5% (v/v) penicillin/streptomycin, (both were supplied by Life Technologies, Paisley, UK) in triple-layered flasks (Nunc™, Thermo Scientific). The supernatant was collected, the cells removed by centrifugation, and the antibody concentrated using Vivaspin columns (Sartorius UK Ltd., UK). The antibodies were isotyped using the IsoStrip mouse monoclonal antibody isotyping kit (Roche Diagnostics, United Kingdom), as per the manufacturer’s instructions. The concentrated antibodies were crudely purified using ammonium sulphate precipitation where 1.4 ml of saturated ammonium sulphate was added to 1 ml of antibody diluted in 1 ml of 0.15 M saline. Following 10 min of mixing, the solution was centrifuged at 4,000 rpm for 10 min, the supernatant discarded and the protein pellet resuspended in 2 ml of saline. This was repeated before final resuspension in 1 ml of saline solution. The antibody concentration was calculated by measuring protein absorbance on a Eppendorf Biophotometer, using saline as a blank. The antibody was aliquoted into working volumes and stored at -80°C for future use.

### Phage display biopanning to identify peptide binders

A commercial Ph.D™ Phage Display Library (Ph.D.-12 New England Biolabs (NEB), Hertfordshire, UK) was used to identify peptide binders using both WCA and EEA of gamma-irradiated MAP strain B4 as the biopanning targets. In total, four rounds of biopanning were performed for each target. Surface biopanning was performed as per the manufacturer’s instructions. Briefly, irradiated MAP WCA or EEA were coated onto two 60 mm Petri dishes at a concentration of approximately 10^9^ cfu/ml or 100 μg/ml of protein prepared in 0.1 M NaHCO_3_ pH 8.6 coating buffer and incubated at 4°C for 24 h with gentle rocking. The Petri dish was blocked with 5 mg/ml BSA in 0.1 M NaHCO_3_ pH8.6 buffer for 1 h at room temperature (RT) before a 1/100 dilution (made in Tris-buffered saline (TBS), 0.1% (v/v) Tween 20, pH 7.5) of the PhD-12 phage library was added with gentle rocking for 1 h. The Petri dish was washed 10 times with TBS 0.1% Tween 20, and the bound phage eluted using 0.2 M Glycine-HCl containing 1 mg/ml BSA. Tris-HCl (pH9.1) was used to neutralise the phages, which were then amplified using *E*. *coli* ER2738. This was followed by centrifugation at 10,000 rpm for 15 min (to remove *E*.*coli* cells) and precipitation of the phage using 20% (w/v) polyethylene glycol prepared in 2.5 M sodium chloride solution. This protocol was repeated using the amplified phage from the first round as the starting library (in place of the PhD-12 library) for the second round and increasing the stringency of the washing procedure, thereby reducing non-specific interactions and enriching the phage library in favour of those capable of binding whole MAP cells.

Solution bio-panning (modified from [22]) was then performed on the third and fourth rounds for the WCA targets only (as it would not have been possible to pellet the EEA antigens by centrifugation). The whole MAP cells were kept free in solution in blocked (TBS-1% BSA) Eppendorf 1.5 ml tubes, rather than being immobilised onto coated petri-dishes (surface biopanning). The remainder of the protocol followed that as outlined for the surface biopanning procedure. For the EEA targets, surface biopanning was used throughout.

**Fig 1:**
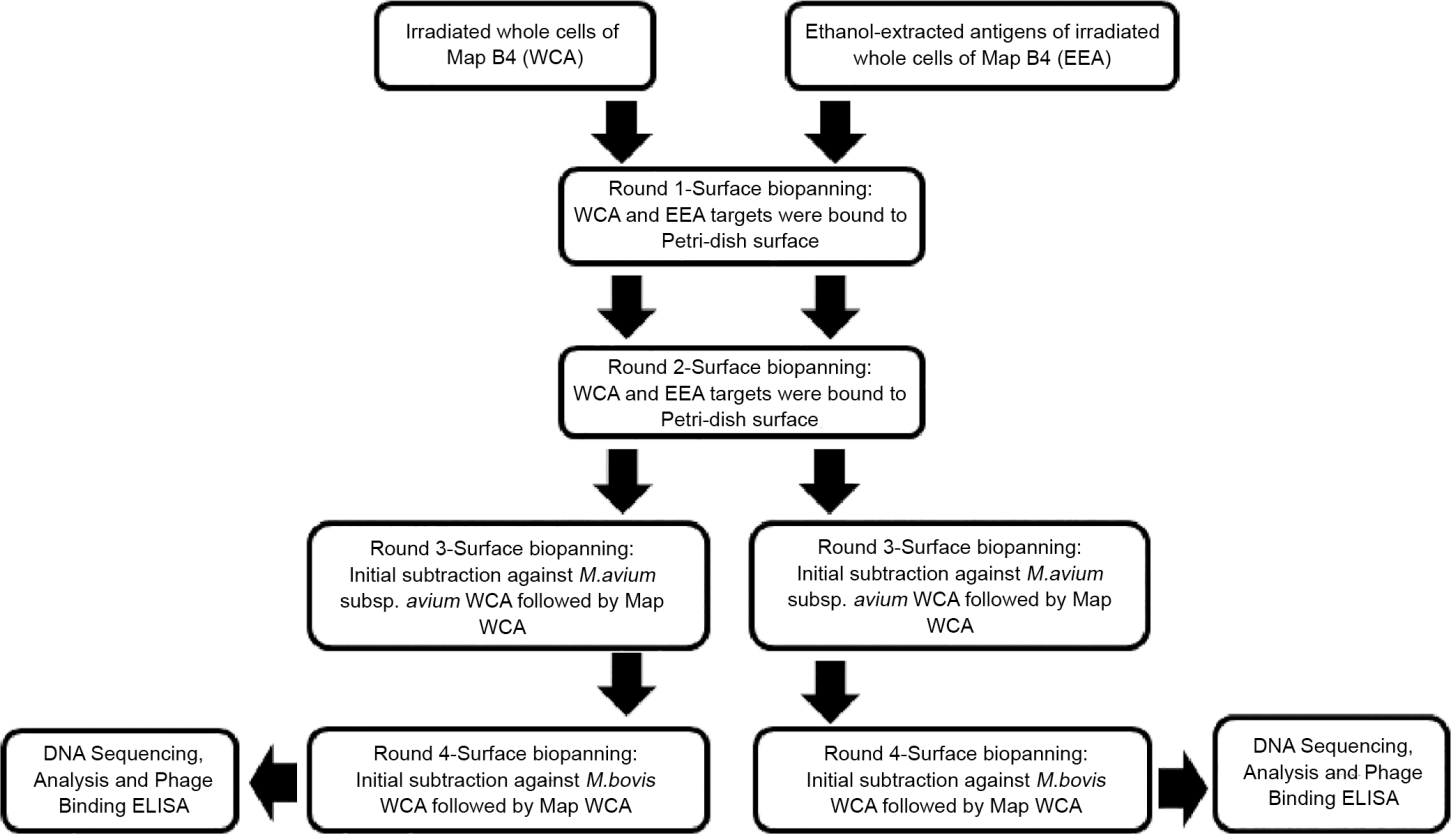
Schematic overview of the phage display biopanning approach taken to identify MAP-specific peptide binders using irradiated MAP B4 whole cell antigen (WCA) and ethanol extracted antigen (EEA) targets.

Two subtraction rounds were incorporated into the last two biopanning rounds for both WCA and EEA MAP targets. For the EEA target, surface-subtraction biopanning was performed whereby a 60 mm Petri dish was coated with 10^6^ cfu/ml irradiated *M*. *bovis* AF2122/97 cells, and another with ~10^8^ cfu/ml irradiated *M*. *avium* subsp. *avium* (MAA) NCTC 13034 cells (suspended in 0.1 M NaHCO_3_ pH 8.6 coating buffer). Two further Petri dishes were coated with 100 μg/ml of MAP EEA. For the WCA target, solution-subtraction biopanning was carried out using blocked Eppendorf tubes containing the same *M*. *bovis* and MAA concentrations in place of the coated petri-dishes. Amplified phage libraries from the second biopanning round were added to the Petri dish or Eppendorf tube containing irradiated *M*. *bovis* cells and incubated for 1 h at RT. The unbound phages were removed and added directly to the Petri dish or Eppendorf tube containing MAP target and incubated for a further 1 h with gentle rocking. The unbound phages were then discarded and the bound phage eluted off using 0.2 M Glycine-HCl with 1 mg/ml BSA pH 2.2 buffer. The rest of the protocol was followed from the NEB PhD-12 phage display library manual. The eluted phage library was amplified and used as the starting library for the fourth round, where it was subtracted against *M*. *avium* subsp. *avium* NCTC 13034. Fig 1 schematically summarises the above process.

### Identification of biopanning peptide sequences

After the fourth round of biopanning, 24 phage plaques were randomly selected, the phage individually amplified in *E*. *coli* ER2738, and the phage DNA extracted and sent for sequencing using _96gIII sequencing primer to DNA Sequencing and Services, University of Dundee, Dundee, Scotland. Corresponding 12-mer peptide sequences were deciphered using FinchTV software (http://www.geospiza.com/Products/finchtv.shtml/) and the ExPASy translate tool (http://web.expasy.org/translate/). Consensus between peptide sequences through the biopanning rounds was analysed using the online ClustalW2 software (http://www.ebi.ac.uk/Tools/msa/clustalw2/). To identify whether these peptide sequences had previously been identified as binding to unrelated target sequences or as plastic binders [23], each of the sequences were also scanned through the online SAROTUP tool (http://immunet.cn/sarotup/index.html, [24]).

Following DNA sequencing, phage expressing different peptide sequences were individually amplified in *E*. *coli* ER2738 and assessed for their ability to bind MAP B4 cells, using the phage binding ELISA as described in the PhD-12 manual. MaxiSorp™ 96-well plates (Nunc™, Thermo Scientific) were coated with 1x10^8^ cfu/ml of irradiated MAP WCA (suspended in 0.1M NaHCO_3_ coating buffer pH8.6). Uncoated wells containing coating buffer only were also included on each plate as control wells, to confirm target-specific binding. Normalised OD readings were calculated by subtracting the OD values of control wells from the OD values of the test wells. Once binding to MAP was confirmed, the most promising peptides were chemically synthesised with a biotinylated N-terminus (by Cambridge Peptides Ltd, Birmingham, UK) to be evaluated for magnetic separation applications.

### Evaluation of novel binders

Selected novel binders from this study plus four monoclonal antibodies kindly provided by Dr Douwe Bakker, Central Veterinary Laboratory of Wageningen University, Lelystad, The Netherlands (Table 1) were initially coated individually onto MyOne™ Tosylactivated Dynabeads® (Life Technologies), as per the manufacturer’s instructions. They were then evaluated singly or in combinations for the magnetic separation of MAP. Initial evaluation of the novel binders was carried out using a dilution containing approximately 10^4^ cfu/ml of MAP strain 806PSS, which was prepared from a stationary phase broth culture and subjected to ultrasonication (pulse mode, 37 kHz for 4 min in ice water ice) to achieve de-clumping [25]. Automated magnetic separation (AMS) was performed using a Dynal BeadRetriever (Life Technologies). DNA was extracted from the MAP cells before and after magnetic separation by boiling for 25 min before being subjected to IS900 PCR [26, 27] to obtain rapid qualitative results indicating degree of MAP capture. The currently used peptide-coated beads, biotin-aMp3 and biotin-aMptD [16], were included for comparative purposes and as a positive control bead.

**Table 1:** Selected MAP binders used to coat MyOne™ tosylactivated Dynabeads® for magnetic separation evaluation.

Bead Number	MAP Binder
**1**	Biotinylated EEA 402 peptide^*a*^
**2**	Biotinylated EEA 405 peptide^*a*^
**3**	Biotinylated EEA 421 peptide^*a*^
**4**	MAb 6G11^*b*^
**5**	MAb 15D10^*b*^
**6**	MAb 10.17.4B(1)^*c*^
**7**	MAb 54.3.1AA ^*c*^
**8**	MAb 51.5.1.1B Gluc ^*c*^
**9**	MAb 54.8.2b1 ^*c*^

^*a*^ Peptide binders identified *via* phage display biopanning during this study and synthesised with biotinylated N-terminus by Cambridge Peptides Limited.

^*b*^ Monoclonal antibodies generated during this project

^*c*^ Monoclonal antibodies supplied by Dr Douwe Bakker, Central Veterinary Institute of Wageningen University, Lelystad, The Netherlands

The capture sensitivity of the best coated beads or combination of coated beads from the initial evaluation was assessed using a range of 10-fold dilutions of MAP strain TB13-008781 (sourced from the Central Veterinary Research Laboratory, Backweston Campus, Celbridge, Co. Kildare, Ireland) ranging from 10^4^−10 cfu/ml. Beads/bead combinations which demonstrated capture sensitivity comparable to or better than that seen with the control biotin-aMp3/biotin-aMptD bead combination, were selected for inclusivity testing using two further MAP strains (TB13-004573 and TB12-017632, both sourced from CVRL), which had been isolated from cattle on two Irish farms. A range of 10-fold dilutions containing 10^4^−10 cfu/ml for each MAP strain were used and the DNA extracted from MAP cells before and after MS. IS900 PCR was performed and the results were compared against those obtained for MAP strains B4 and TB13-008781. A greater range of MAP strains was used for inclusivity testing at a later date using the phage amplification assay as the endpoint detection method.

### Evaluation of binder specificity

To determine the percentage of non-specific capture by the variously coated magnetic beads, live stationary cultures of a range of *Mycobacterium* spp. (*M*. *avium* subsp. *avium* NCTC 13034, *M*. *bovis* BCG NCTC 5692, *M*. *terrae* NCTC 10856, *M*. *kansasii* NCTC 10268, *M*. *marinum* NCTC 2275, *M*. *smegmatis* mc^2^155, *M*. *intracellulare* NCTC 10425, *M*. *fortuitum* NCTC 10394, *M*. *hiberniae* (field isolate) and *M*. *xenopi* NCTC 10042) were diluted to a concentration of 10^4^−10^3^ cfu/ml and subjected to magnetic separation. Numbers of each *Mycobacterium* sp. before and after MS were determined by plating 100 μl of the initial cell suspensions and the bead suspensions after MS on Middlebrook 7H10/OADC agar (both Difco). The agar plates were wrapped in Duraseal Laboratory Film (Diversified Biotech, MA, USA) and incubated at the respective optimal temperature of the *Mycobacterium* sp. being tested until countable colonies were visible. Percentage capture of each of the *Mycobacterium* spp. was determined by expressing the count after MS as a percentage of the count before MS. An overall mean percentage non-specific capture and standard error for each bead/bead combination was calculated using results obtained for all ten *Mycobacterium* spp.

Additionally, the non-specific capture of raw milk microflora by selected beads/bead combinations was evaluated, as well as the ability to control the outgrowth of these non-specifically bound/adhering milk microorganisms by the addition of antibiotics to 7H9/OADC broth medium. Following MS, the final 1 ml sample volume was split three-ways to inoculate 10 ml of 7H9/OADC broth containing the commercially available antibiotic supplements NOA [28] (Nystatin 50,000 IU/l, Oxacillin 2mg/l and Aztreonam 30 mg/l; Abtek Biologicals Ltd, Liverpool, UK), PANTA (Polymyxin B 40,000 IU/l, Amphotericin B 4,000μg/l, Nalidixic acid 16,000 μg/l, Trimethoprim 4,000μg/l, and Azlocillin 4,000μg/l; Becton Dickinson, Oxford, UK) and antibiotic-free 7H9/OADC broth as a control. All broths were incubated at 37°C and absorbance measurements at OD_600nm_ (measured using Biowave CO8000 Density meter, Biochrom Ltd., Cambridge, UK) were recorded periodically as a measure of microbial growth over time.

## Results

### Antibody production

**Fig 2:**
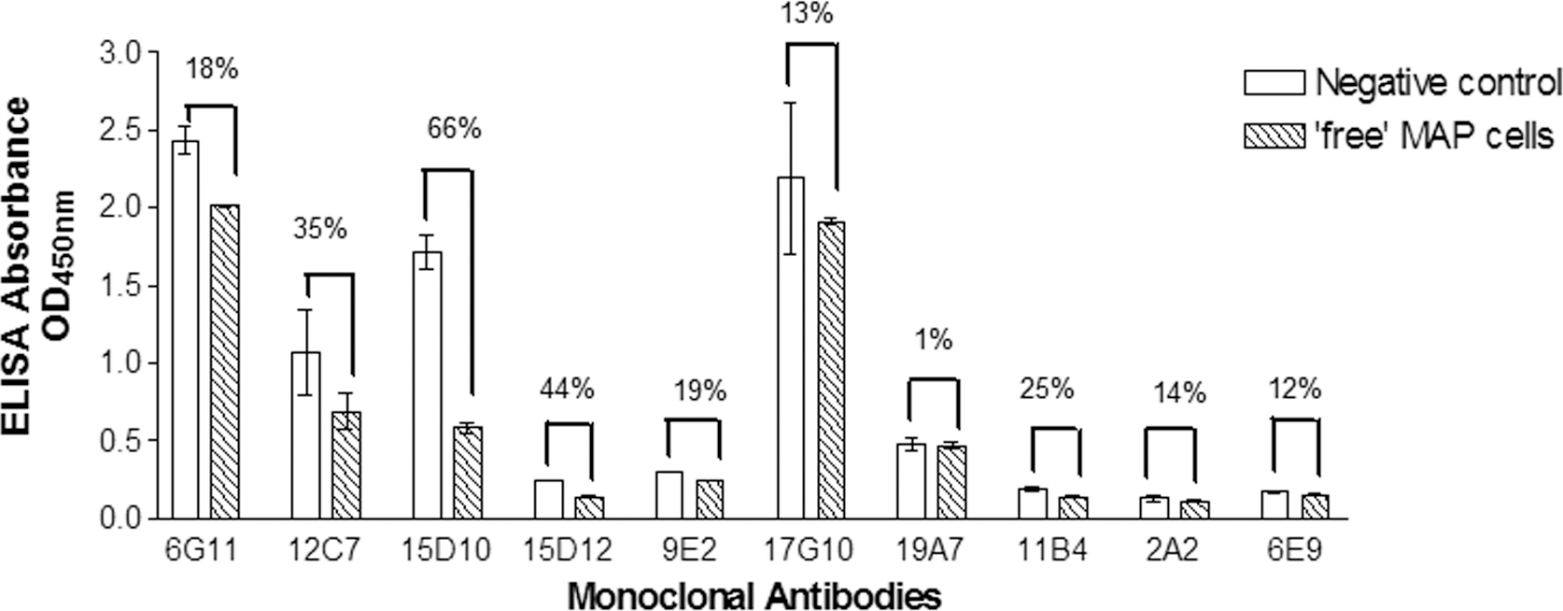
Competitive ELISA results obtained for WCA and EEA generated monoclonal antibodies. Bacterial suspension of irradiated MAP was added wells as ‘free MAP cells’ prior to the addition of the antibody. Negative control wells received assay buffer followed by the antibody. A decrease in absorbance values in the presence of ‘free MAP cells’ over negative control wells is indicative of MAP binding. Figure depicts the mean absorbance values (n = 3) ± standard error. Mean % binding inhibition was calculated based on the difference in the % binding of the antibody to the attached MAP cells in the presence and absence of ‘free MAP cells’, where negative control wells represent 100% binding.

Mice immunised with MAP WCA and EEA elicited immune responses after just two immunisations, evidenced by binding of whole MAP cells by direct and competitive ELISA. This positive binding response increased further following two subsequent immunisations; a high antibody titre of 1:8,000 was obtained for both immunogens by competitive ELISA. A total of 2308 hybridoma supernatants (706 from WCA immunisation and 1602 from EEA immunisation) were screened by direct ELISA, of which 47 demonstrated binding to whole MAP cells. Of these, only two cell lines from the WCA immunogen (6G11 and 12C7) and eight from the EEA immunogen (15D10, 15D12, 9E2, 17G10, 19A7, 11B4, 2A2 and 6E9) showed inhibition of binding by competitive ELISA (Fig 2). Monoclonal antibody cell lines 6G11 from the WCA fusion and 15D10 from the EEA fusion were selected for further evaluation based on their competitive ELISA results, with ~18% and ~66% binding inhibition respectively (% binding inhibition was calculated by subtracting the % binding obtained in the presence of ‘free MAP cells’ from 100% binding observed in the negative control wells; % binding refers to antibody bound to attached MAP cells on the well surface). Following antibody purification, both 6G11 and 15D10 monoclonal antibodies were identified as IgM antibodies using the IsoStrip kit.

### Phage display biopanning

After four rounds of biopanning, 24 clones from each biopanning target were selected at random for sequencing; nine different peptide sequences were identified from the WCA biopanning and eight peptide sequences from the EEA biopanning. With both biopanning targets, the same peptide sequence was repeated several times, while others differed by only one or two amino acids, demonstrating a high level of consensus among the peptide sequences identified by each target (Table 2). When checked using the SAROTUP tool, none of these peptide sequences had previously been shown to be binders for other unrelated targets or as binding to plastic. The WCA target identified four sequences which demonstrated a low level of binding to the whole MAP cells (WCA MAP target), resulting in weak ELISA signals (normalised OD_450nm_ values < 0.5). In contrast, all eight peptide sequences identified from the EEA target demonstrated a high degree of binding to whole MAP cells, resulting in strong ELISA signals (normalised OD_450nm_ values > 1). Three of the EEA peptide sequences (EEA402, EEA405 and EEA421) were selected to be chemically synthesised (with a biotinylated N-terminus), based on their ability to bind whole MAP cells, and subsequently assessed for MS application.

**Table 2:** Summary of outcomes of phage display biopanning of MAP WCA and EEA with sequences selected for MS evaluation highlighted.

Biopanning target	Clone identifier	Peptide sequence	Frequency^*a*^	Binding to whole MAP cells^*b*^	ELISA signal^*c*^
WCA	401	WPAHFIISPVE	1/17	Yes	Weak
	402	PKLLKVV-QNPI	1/17	No	
	403	VWHDFRQWWQPS	8/17	No	
	404	SGVYKVAYDWAH	2/17	Yes	Weak
	407	AETVESCFSKIP	1/17	Yes	Weak
	408	DSQFNKYSIATV	1/17	No	
	411	GLHTSATNLYLH	1/17	No	
	412	GLSVSREVPDKV	1/17	Yes	Weak
	414	VWHDFRQWWQPY	1/17	No	
EEA	**402**	**VWHVGFLRQLL**	**1/15**	**Yes**	**Strong**
	403	RKVKRRLLVSKL	1/15	Yes	Strong
	404	AENQVRVRNSLD	1/15	Yes	Strong
	**405**	**DSVMPLKVPYIP**	**7/15**	**Yes**	**Strong**
	407	RKVKRRPRVSNL	2/15	Yes	Strong
	419	RKVKRRPRVLNL	1/15	Yes	Strong
	**421**	**RKVKRRPHVSNL**	**1/15**	**Yes**	**Strong**
	424	STGSVRGQHAKG	1/15	Yes	Weak

^*a*^ number of phage clones with the same peptide sequence per total number of phage clones sequenced

^*b*^ binding to whole MAP cells tested by direct ELISA

^*c*^ Strength of ELISA signals obtained after the absorbance values were normalised using uncoated control wells. Normalised absorbance values > 1 were considered ‘strong’, values < 0.5 ‘weak’, and values between 0.5 and 1 were considered ‘medium’.

### Evaluation of monoclonal antibodies and peptides for magnetic separation application

**Fig 3:**
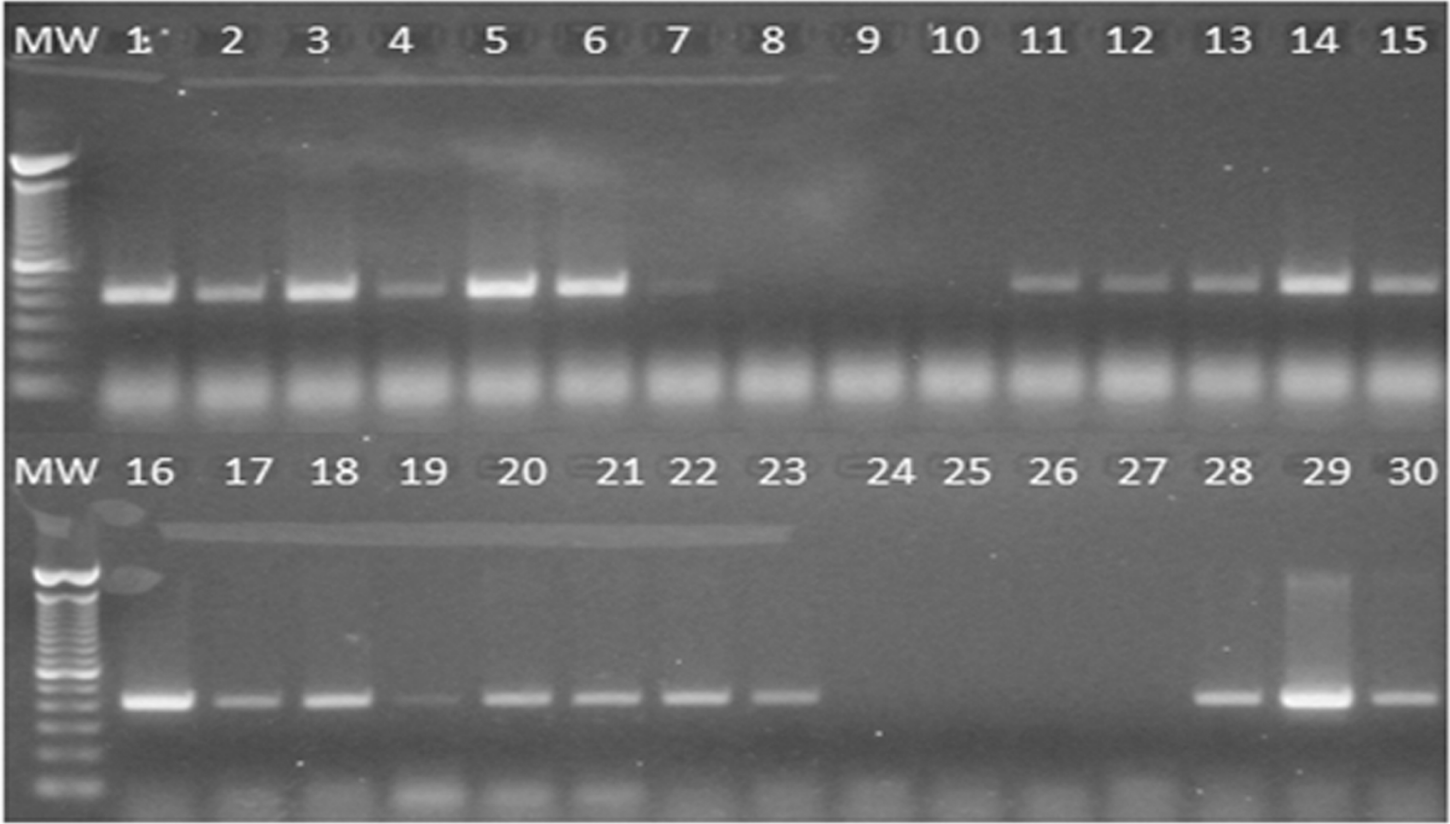
Evaluation of differently coated MyOne™ Tosylactivated Dynabeads® for magnetic separation of MAP 806PSS from a PBS suspension containing approximately 10^4^ CFU/ml. Beads were coated separately with different MAP binders (see Table 1) and then evaluated for MS individually (10 μl of beads per ml of sample) and as 50:50 combinations (5 μl of each bead type per ml of sample). Ability to capture MAP was evaluated by IS900 PCR; the expected PCR product size was 394 bp and intensity of signal was used to assess degree of MAP capture. Key: Lanes 1 and 16, MAP 806PSS suspension before MS; Lane 2–10, coated beads 1–9 tested individually; Lane 11, beads 1 and 2; Lane 12, beads 1 and 3; Lane 13, beads 2 and 3; Lane 14, beads 1 and 4;. Lane 15, biotinylated aMp3 and aMptD peptide-coated beads (control) currently employed for MAP capture at QUB; Lane 17, beads 2 and 4; Lane 18, beads 3 and 4; Lane 19, beads 1 and 5; Lane 20, beads 2 and 5; Lane 21, beads 3 and 5; Lane 22, beads 4 and 5; Lane 23, beads 1 and 7; Lane 24, beads 2 and 7; Lane 25, beads 3 and 7; Lane 26, beads 1 and 6; Lane 27, beads 2 and 6; Lane 28, beads 3 and 6; Lane 29, beads 4 and 7. Lane 30, biotinylated aMp3 and aMptD peptide-coated beads (control). Lanes MW indicate a 100 bp DNA ladder (TrackIt™, Life Technologies).

MyOne™ Tosylactivated Dynabeads® coated individually with monoclonal antibodies 6G11, 15D10 and 10.17.4(B) 1 as well as the synthetized peptides biotin-EEA402, biotin-EEA405 and biotin-EEA421 demonstrated binding of 10^4^ cfu/ml of whole MAP cells (Fig 3). When PCR band intensity was quantified numerically using UVP Doc-ItLS Image analysis Software (Ultra Violet Products Ltd, Cambridge, UK) an I-Max value (maximum band intensity achieved per lane), was calculated. Using the I-max value as a guide, monoclonal antibodies 6G11 and 15D10 and peptide biotin-EEA405 individually coated beads generated PCR products which were comparable to that observed before MS (I-Max = 230), with band intensity values of 242, 226 and 169, respectively. Multiple bead combinations (50:50 mix of individually-coated beads) were also assessed for their ability to capture 10^4^ cfu/ml of MAP. Based on the numerical value attributed to the PCR band intensity, the bead combinations which most closely reflected that seen before MS (lane 1; I-Max = 230) were; MAbs 6G11 and 54.3.1AA (lane 29; I-Max = 253); MAb 6G11 and biotin-EEA402 (lane 14; I-Max = 213); MAb 6G11 and biotin-EEA421 (lane 18; I-Max = 202); MAb 10.17.4(B)1 and biotin-EEA421 (lane 28; I-Max = 194); MAbs 6G11 and 15D10 (lane 22; I-Max = 192); MAb 15D10 and biotin-EEA405 (lane20; I-Max = 186) and MAb 15D10 and biotin-EEA421 (lane21; I-Max = 181) (Fig 3).

**Fig 4:**
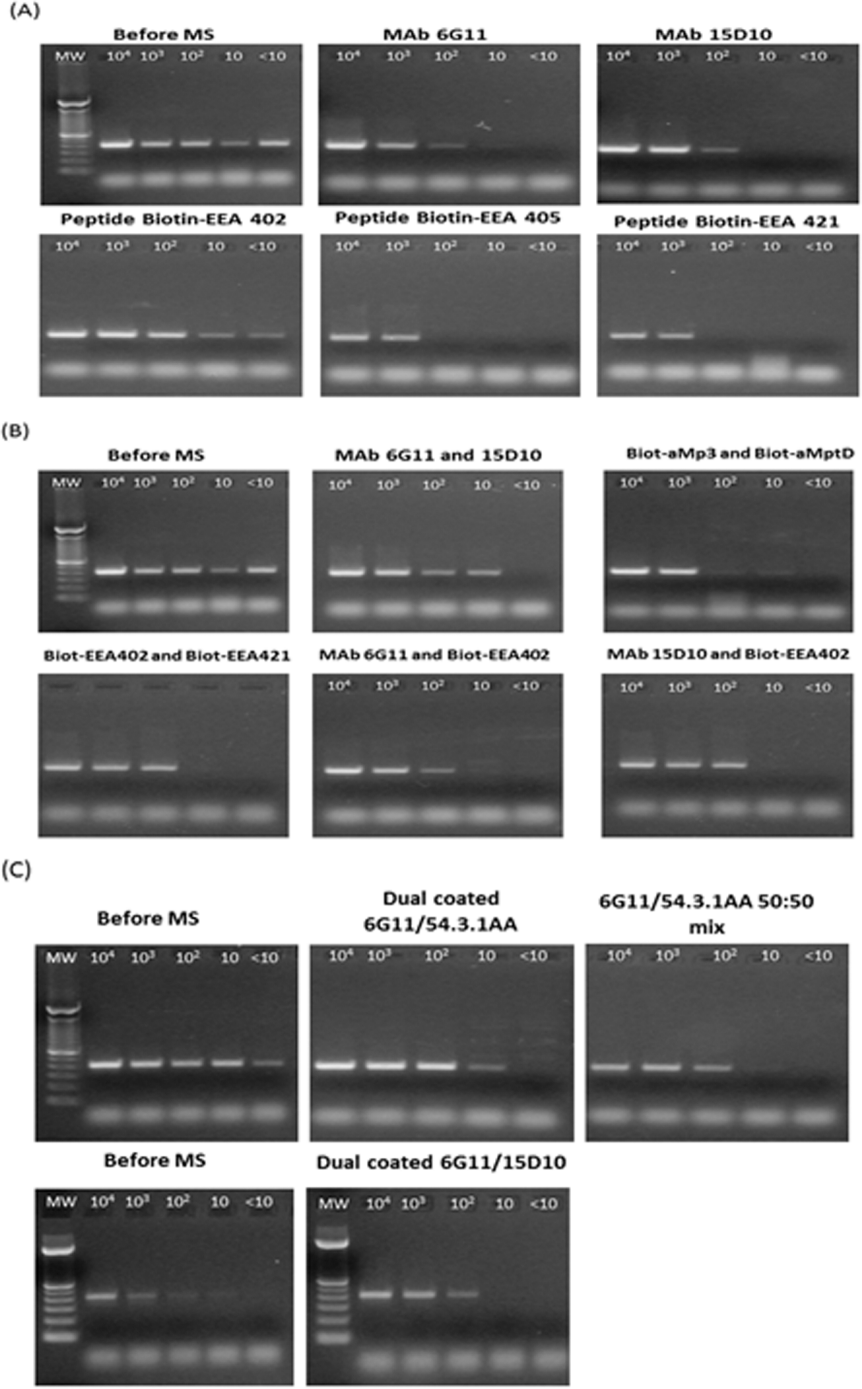
MAP capture sensitivity of magnetic beads coated with monoclonal antibodies and peptides to capture whole cells of MAP strain TB13-008781 from PBS dilutions containing approx. 10^4^−10 cfu/ml when used (A) individually, (B) as 50:50 bead combinations, or (C) as dually-coated beads. DNA extracted from the bacterial suspensions before MS was used as a control. The expected IS900 PCR product is 394 bp. Lane MW indicates a 100 bp DNA ladder (TrackIt™, Life Technologies).

Following initial evaluation, the capture sensitivity of the beads and bead combinations which had demonstrated the highest degree of MAP capture were assessed using MAP (isolate TB13-008781) at a range of concentrations (10−10^4^ cfu/ml). The capture sensitivity of the novel MAP binders was compared against that seen before magnetic separation and that seen by the currently used biotin-aMp3 and biotin-aMptD peptide-coated beads. Beads coated individually with MAb 6G11, MAb 15D10 and biotin-EEA402 proved to have the highest capture sensitivity, with biotin-EEA402 beads capturing MAP from suspensions containing approximately 10 cfu/ml (Fig 4A). Beads coated with peptides biotin-EEA405 and biotin-EEA421 were the least sensitive, only capturing MAP cells from suspensions containing at least 10^3^ cfu/ml. Of the bead combinations tested, the most promising were MAbs 6G11 and 15D10 (Fig 4B), which were able to capture MAP cells when >10 cfu/ml were present. Bead combinations MAb 6G11 and biotin-EEA402 and MAb 15D10 and biotin-EEA402 had comparable capture sensitivity to that seen by the control peptide-coated beads (biotin-aMp3 and biotin-aMptD) of ~ 10^3^−10^2^ cfu/ml. However, these three bead combinations showed greater MAP capture at each of the tested cell concentrations, evidenced by higher PCR band intensities than those that were observed for the biotin-aMp3 and aMptD combination; the band intensities observed for the novel coated beads/bead combinations more closely resembled those seen for the MAP suspensions before MS. A further promising bead combination was MAb 6G11 and MAb 54.3.1AA (Fig 4C). When used as a 50:50 mix of individually coated beads, they were able to capture MAP from suspensions containing 10 cfu/ml. These beads were also assessed as dually coated beads (beads coated with the two antibodies simultaneously); however dual coating did not appear to enhance capture sensitivity. Similarly, the degree of capture was the same when beads were either dual coated with MAbs 6G11 and 15D10 or individually coated and used as a 50:50 mix.

**Fig 5:**
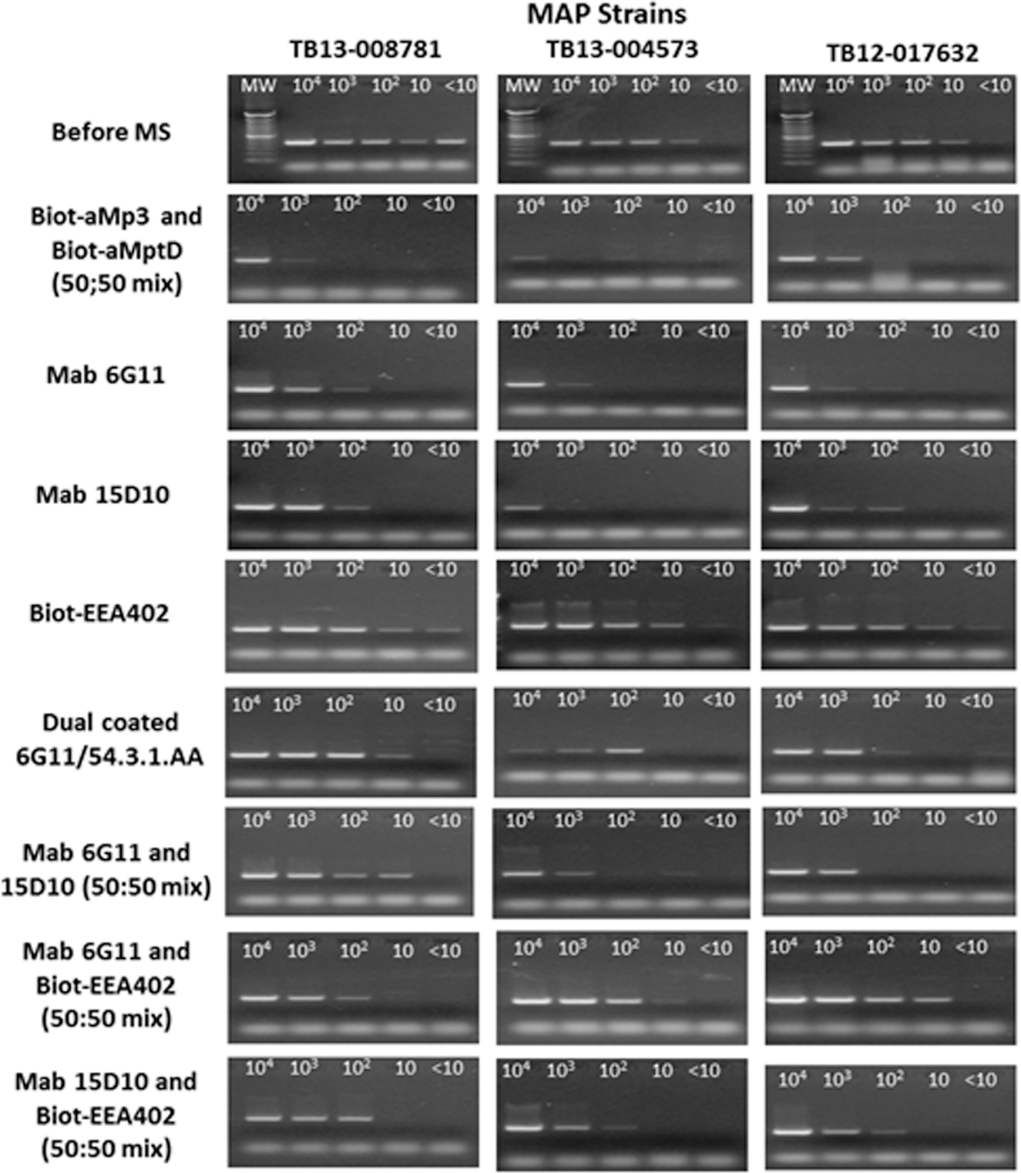
Inclusivity testing with the ‘most promising’ beads or bead combinations (50:50 mix or dually coated) to capture three different MAP strains (TB13-008781, TB13-004573, TB12-017632) from PBS dilutions containing from 10^4^−10 cfu/ml. DNA extracted from the bacterial suspensions before MS was used as a control. The expected IS900 PCR product is 394 bp. Lane MW indicate a 100 bp DNA ladder (TrackIt™, Life Technologies).

Those beads or bead combinations which showed greater capture sensitivity than shown by the current aMp3/aMptD peptide-coated beads were subjected to inclusivity testing using a further two MAP isolates. Beads coated with biotin-EEA402 demonstrated MAP capture in solutions containing < 10 cfu/ml for each of the strains (Fig 5). Dually coated MAb 6G11 and 54.3.1AA beads were capable of binding MAP cells in solutions containing approx. 10 cfu/ml. MAbs 6G11 and 15D10 performed similarly, capturing MAP from solutions containing between 10 and 10^2^ cfu/ml. MAP capture improved further when MAbs 6G11 and 15D10 were used as a 50:50 mix. Overall, the novel coated beads/bead combinations all performed better than the currently used biotinylated aMp3 and aMptD peptide-coated beads.

### Evaluation of MS specificity

**Fig 6:**
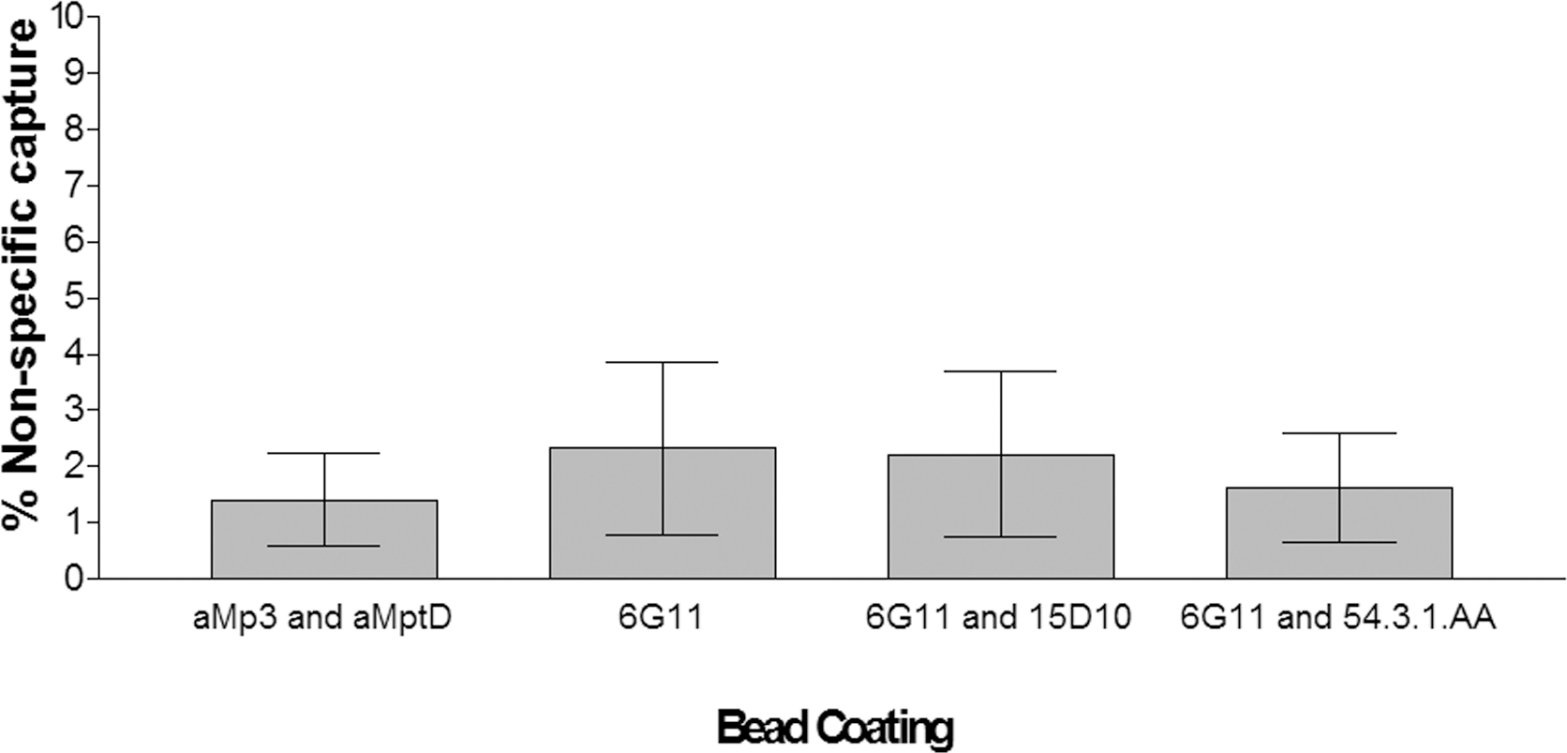
Percentage non-specific capture of a range of *Mycobacterium* spp. (detailed in Materials and Methods) by variously coated Dynabeads. Figure depicts mean percentage capture ± standard error of results for ten different *Mycobacterium* spp. captured by each bead or bead combination: MAb 6G11 coated beads; beads coated individually with MAb 6G11 and MAb 15D10 and used as 50:50 mix; beads dually coated with MAb 6G11 and MAb 54.3.1.AA; beads coated individually with biotinylated aMp3 and aMptD peptides and used as 50:50 mix (control).

Due to the number of promising bead/bead combinations, the capture specificity of the coated beads was initially assessed using three non-target *Mycobacterium* spp. (*M*. *avium* subsp. *avium*, *M*. *bovis* BCG and *M*. *terrae*). Beads or bead combinations which used the biotin-EEA402 peptide showed a high degree of cross-reactivity with each of these species (mean percentage capture was ~40%), and therefore was eliminated from further specificity testing. Beads coated with MAb 6G11, the bead combination MAb 6G11 and 15D10 (50:50 mix), and dually-coated MAb 6G11 and 54.3.1AA beads demonstrated a low percentage of non-specific capture (ranging from 0.75–15% capture, with *M*. *bovis* BCG giving the highest percentage capture for all bead types). The coated beads were then tested against a further seven non-target *Mycobacterium* spp. (*M*. *intracellulare*, *M*. *marinum*, *M*. *xenopi*, *M*. *hiberniae*, *M*. *kansasii*, *M*. *fortuitum* and *M*. *smegmatis*). A mean percentage of non-specific capture for the ten *Mycobacterium* spp. was calculated for each of these coated bead/bead combinations (Fig 6). The currently used aMp3/aMptD peptide-coated beads were included for comparative purposes. When beads coated with MAb 6G11 were used on their own or in a 50:50 mix with MAb15D10-coated beads a mean percentage non-specific capture of 2.3% was achieved. Dually coated MAb 6G11 and 54.3.1.AA beads performed the best, and were comparable to the currently used aMp3/aMptD peptide-coated beads, with a mean percentage non-specific capture of approx.1.6% (Fig 6).

### Evaluation of non-specific binding of raw milk microflora by selected beads

**Fig 7:**
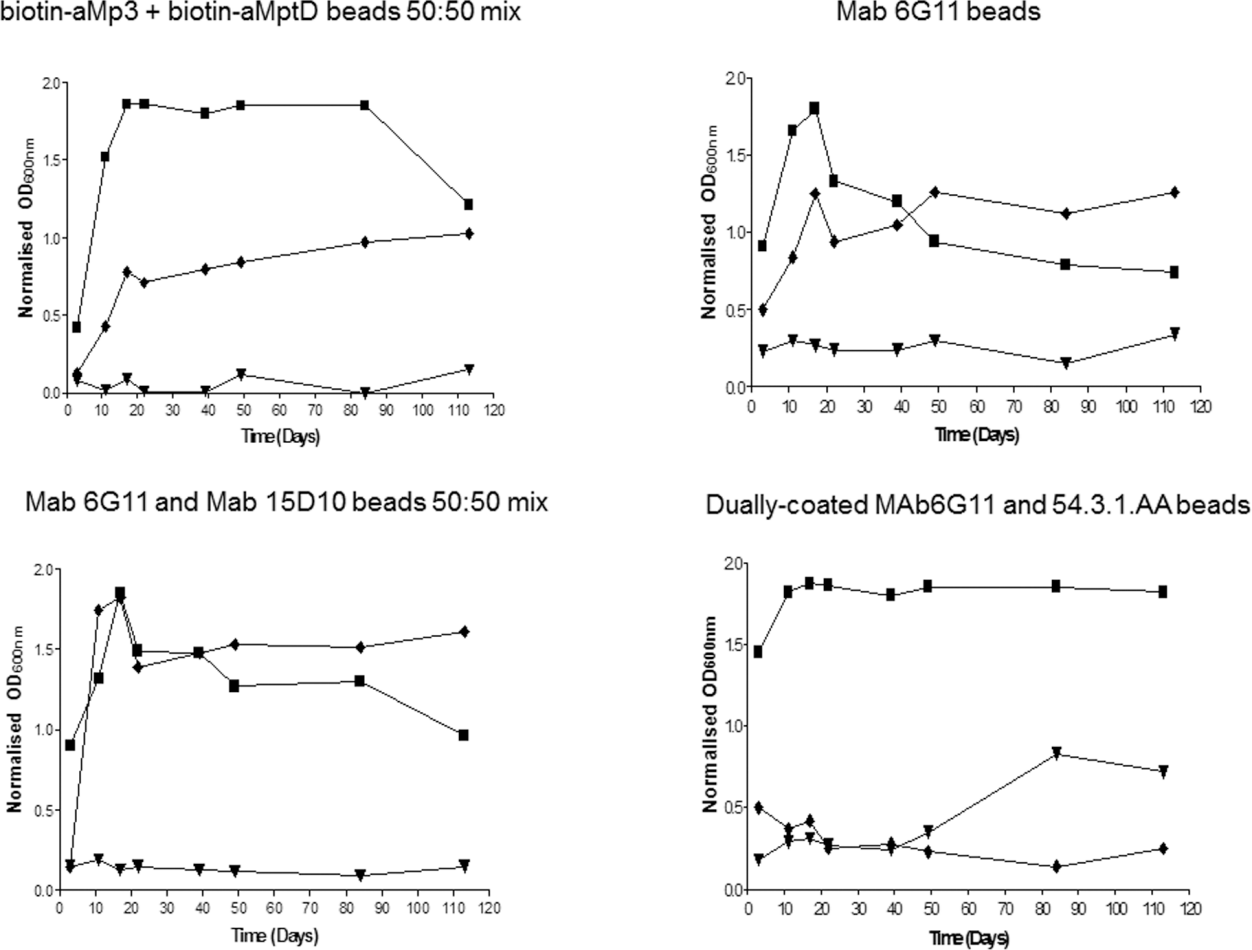
Outgrowth of milk bacteria non-specifically adhering to four types of coated magnetic beads in Middlebrook 7H9 OADC broth without (■) and with antibiotic supplements (NOA ◆ and PANTA ▼) after MS was applied to raw milk.

The three most promising coated beads, plus the control biotin-aMp3 and biotin-aMptD peptide-coated beads, were also assessed for their non-specific binding of raw milk microflora (Fig 7). An increase in OD was observed for each of the bead types after 3 days of incubation in antibiotic-free broth medium. The current peptide-coated beads showed the lowest degree of non-specific binding to raw milk microflora, followed by MAb 6G11 beads when used singly or in combination with MAb 15D10, which both performed similarly. The highest degree of non-specific binding was seen for the dually coated MAb 6G11 and 54.3.1AA beads (Fig 7). We investigated the ability of two different antibiotic supplements, NOA and PANTA, to combat outgrowth of adhering milk bacteria in Middlebrook 7H9 OADC broth. Broths supplemented with NOA showed reduced growth in the first few days of incubation compared with antibiotic-free broth, however after approximately 10 days of incubation, some of the broths had reached similar OD readings as seen by the antibiotic-free broth cultures (Fig 7). In contrast, broths supplemented with PANTA showed the greatest degree of growth suppression; the maximum OD 600nm value achieved in the presence of PANTA was 0.3 for most broths (with the exception of broth from dually coated MAb6G11 and 54.3.1.AA beads) after 113 days. Thus, supplementing broth with PANTA would provide the best environment to support the growth of MAP extracted from raw milk by MS.

## Discussion

The objectives of this study were firstly, to generate monoclonal antibodies and phage display-derived peptide binders which were capable of binding whole cells of MAP, and secondly, to assess these novel binders for their potential application to capture and concentrate MAP by magnetic separation. Magnetic separation protocols have been extensively applied to the isolation of a wide variety of bacteria across a large range of food, clinical and environmental matrices, each of which has produced varying degrees of success. For example, magnetic separation protocols optimised for the capture of *M*. *bovis* found that the combination of a monoclonal antibody and a synthetic peptide binder demonstrated the greatest degree of assay sensitivity and specificity [19]. Other studies, found that when beads were coated with either polyclonal antibodies or synthetic peptides a similar level of capture for *Salmonella* Typhimurium was observed [29, 30]. Based on the varying degrees of success with peptides, monoclonal and polyclonal antibodies for magnetic separation, it was important to investigate which combinations of antibodies and/or peptides would improve the sensitivity and specificity for the magnetic separation of MAP.

In order to generate MAP-specific binders, it is critical that the cell surface is not compromised and retains the integrity of its cell surface antigens. Previous studies have often employed heat treatments to inactivate MAP cells; however this method may denature heat-labile surface proteins, distorting their conformation and thereby affecting immune and/or phage-peptide recognition. In the present study gamma irradiation was employed to inactivate the MAP cells, by destroying the cellular DNA whilst maintaining the surface protein structure with minimal or no damage. In order to ensure the greatest chance of success in binder generation both whole cell antigen (WCA) and a suspension of the cell surface antigens (EEA), extracted with alcohol and vortexing, were employed as immunogens and phage-display biopanning targets. Eda *et al*. [20] postulated that by extracting these antigens from the cell surface a high percentage will be species-specific rather than generic mycobacterial cell wall components. Consequently this approach may aid in the generation of highly specific binders for MAP.

Previously, a combination of surface and solution biopanning steps was found to increase the specificity of *Listeria* binders compared to several rounds of surface biopanning alone [31], so this approach was adopted for MAP. By using a combination of surface and solution biopanning, the presentation of the target changes from two-dimensional to three-dimensional, thereby increasing the potential number of surface antigens available for phage binding. This is also true of using EEA rather than WCA. Results obtained during this study found that EEA targets identified peptide sequences which exhibited a higher binding affinity for MAP over those identified by the WCA target as seen by ELISA; a finding which had also been seen in a previous study using *M*. *bovis* EEA biopanning targets [19].

To generate monoclonal antibodies which were specific for MAP, we applied the same dual approach using both WCA and EEA of MAP to initiate an immune response in mice. Both WCA and EEA of MAP were found to be highly immunogenic, resulting in equally high murine antibody titres. Despite receiving five immunisations over a five month period, the majority of the antibodies obtained were classified as IgM, although some IgG antibodies were also present, a finding which has been noted in previous studies [32, 33]. WCA and EEA immunogens successfully generated antibodies which were highly specific and sensitive for binding whole cells of MAP, on both ELISA and magnetic separation.

All of the novel MAP binders generated in this study were capable of binding MAP with varying levels of sensitivity and specificity. However, two bead types stood out during MS evaluations, namely biotin-EEA402 coated beads and dually coated MAbs 6G11 and 15D10 beads, as having superior performance (6G11 and 54.3.1AA were discounted based on their non-specific capture of milk microflora). Magnetic separation using biotinylated EEA402 coated beads proved to be much more sensitive at capturing whole MAP cells than any of the other bead combinations tested as well as that seen by the currently used PMS (biotinylated aMp3 and aMptD peptides) assay. However, EEA402 beads were not found to be specific for MAP, as they demonstrated a high percentage capture of a range of other *Mycobacterium* spp. It is possible that this peptide is binding to a more generic mycobacterial surface antigen rather than a species-specific antigen. As the majority of the EEA preparation is comprised of cell surface carbohydrates [20], it is likely that the EEA402 peptide binds to a carbohydrate target on the cell surface, one of which is common to most *Mycobacterium* spp. As this peptide ligand was not found to be MAP-specific, it would not be suitable for use in the combined MS-culture or MS-phage assays. Instead, combining biotin-EEA402 magnetic capture with a PCR with high specificity for MAP would be a better prospect.

Due to the demonstrated specificity of monoclonal antibodies 6G11 and 15D10 generated during this study, the application of an IMS protocol employing coated 6G11 and 15D10 magnetic beads may be much more widely applied. MAP capture was maintained when the beads were dually-coated with 6G11 and 15D10 or individually coated and used as a 50:50 mix, thus MyOne™ Tosylactivated Dynabeads® either singly coated or dually-coated with monoclonal antibodies 6G11 and 15D10 were selected as the optimal binder combination for use in the combined MS- culture and MS-phage assays. Additionally, the potential application of IMS-culture from raw milk was demonstrated. Low numbers of carryover bacteria are inevitable. However, this study has shown that supplementation of the culture medium with PANTA successfully combatted the growth of adhering milk microorganisms over the 4 month incubation period. The dually coated beads for IMS could be used in combination with the phage amplification assay, which is based on the infection and lysis of MAP cells by the D29 bacteriophage. This bacteriophage has a broad mycobacterial host range, and hence assay specificity must come from the magnetic separation protocol. The phage assay had originally been used for the detection of *M*. *tuberculosis* from clinical samples but was later modified for MAP detection based on the specific MAP burst time. Cell lysis is dependent on the host’s generation time, and therefore the burst time may be different for each bacterial species. As many of the other mycobacterial species have shorter burst times than MAP (including *Mycobacterium avium* subsp. *avium*); the modified phage assay should eliminate those species during the virucidal treatment. However, the burst times for MAP and *M*. *bovis* are similar and therefore cross-reactivity with *M*. *bovis* cells during magnetic separation could result in plaque formation during the phage assay. In the case of samples taken from herds where bovine TB is suspected a further molecular confirmatory test would need to be included. Further optimisation of the IMS-phage assay is currently underway.

## Conclusions

Novel monoclonal antibody and peptide binders specific for MAP were successfully generated, and two separate MS approaches are suggested for the isolation of MAP cells: (1) PMS using biotin-EEA402 peptide-coated beads and (2) IMS using dually-coated monoclonal antibodies 6G11 and 15D10 beads. However, successful application of each will be dependent on the endpoint detection method selected; PCR in the case of PMS and culture or phage amplification assay in the case of IMS. Both types of coated beads were found to improve the sensitivity of MAP capture compared to the currently used aMp3/aMptD peptide-coated beads, however only the IMS assay maintained >98% assay specificity.
